# Dry Eye and Phacoemulsification Cataract Surgery: A Systematic Review and Meta-Analysis

**DOI:** 10.3389/fmed.2021.649030

**Published:** 2021-07-08

**Authors:** Qiang Lu, Yi Lu, Xiangjia Zhu

**Affiliations:** ^1^Eye Institute and Department of Ophthalmology, Eye & ENT Hospital, Fudan University, Shanghai, China; ^2^NHC Key Laboratory of Myopia (Fudan University), Shanghai, China; ^3^Key Laboratory of Myopia, Chinese Academy of Medical Sciences, Shanghai, China; ^4^Shanghai Key Laboratory of Visual Impariment and Restoration, Shanghai, China

**Keywords:** phacoemulsification, Schirmer test, corneal fluorescein staining, tear break-up time, subjective symptoms, diabetes, meibomian gland dysfunction, dry eyes

## Abstract

**Purpose:** To evaluate whether dry eye deteriorates after phacoemulsification cataract surgery, and to explore the influential factors.

**Methods:** Studies published before February 2020 indexed on PubMed and the Cochrane Central Register of Controlled Trials were retrieved. A meta-analysis, including meta-regression, a sensitivity analysis, and a subgroup analysis, were performed.

**Results:** Twenty studies with 2,247 eyes were included in the meta-analysis, dry eye-related parameters were investigated preoperatively and 1 month postoperatively. Patients with pre-existing meibomian gland dysfunction (MGD) had worsened subjective symptoms of dry eye (1.31, 95% confidence interval (CI) [0.66, 1.95], *P* < 0.0001), a reduced tear break-up time (BUT) (−2.27, 95% CI [−2.66, −1.88], *P* < 0.0001), and a worse corneal fluorescein staining (CFS) score (0.75, 95% CI [0.5, 1.0], *P* < 0.0001) after phacoemulsification cataract surgery, whereas in the general population, the subjective symptoms score and CFS remained unchanged and BUT decreased slightly after surgery. Patients without diabetes showed significantly reduced total tear secretion after phacoemulsification cataract surgery (−1.25, 95% CI [−1.62, −0.88], *P* < 0.0001).

**Conclusion:** Dry eye generally remained unchanged 1 month after phacoemulsification cataract surgery. Notably, worsened symptoms and signs of dry eye were observed more frequently in patients with pre-existing MGD. Patients without diabetes were more susceptible to reduced tearing postoperatively.

**Clinical Trial Registration:** Identifier: PERSPERO (2020: CRD42020203316).

## Introduction

Dry eye is one of the commonest complaints reported in ophthalmology clinics, currently accounting for 17–25% of outpatient visits (1). The prevalence of dry eye disease (DED) ranges from 6 to 34% (2–6), and it can lead to a constellation of clinical signs and symptoms, including ocular fatigue, discharge, foreign body sensation, and epiphora (7).

Although there are many potentially DED-inducing factors both intraoperatively and postoperatively (7–11), whether cataract surgery is a risk factor for DED remains controversial. Several studies report that patients remain unsatisfied and disturbed by postoperative DED for long periods (12, 13), whereas others consider DED after cataract surgery to be a manifestation of the transitory impairment of the ocular surface, and that the damaging effect tapers off within 1–3 months (7, 13–17). Other studies have suggested that the ocular surface is improved after cataract surgery, which is thought to correlate with the postoperative use of eye drops, reduced eye rubbing, and adequate blinking (18, 19). Meibomian gland dysfunction (MGD) is an important etiological factor for DED and is also responsible for postoperative ocular discomfort and dry eye (7, 20).

Phacoemulsification, with its small incision and satisfactory safety performance, have become the major procedure in regular cataract surgery. To understand better whether phacoemulsification cataract surgery induces or aggravates postoperative dry eye, we undertook a systematic review and meta-analysis of the published literature on DED-relevant parameters, including questionnaires on subjective symptoms, tear break-up time (BUT), corneal fluorescein staining (CFS), and the Schirmer I test. We paid special attention to potential DED-influencing factors, such as pre-existing MGD (7), diabetes mellitus (DM) (21, 22), the preoperative status of the ocular surface, the incision size (9, 23), and the country of origin.

## Methods

### Search Strategy

The protocol was registered in PROSPERO (2020: CRD42020203316). A systematic search was conducted of PubMed and the Cochrane Central Register of Controlled Trials (CENTRAL) for studies reporting DED-related parameters measured with both preoperative and postoperative tests. The search terms were: phacoemulsification, cataract surgery, ocular surface, dry eye, questionnaires, tear film, tear stability, tear secretion, tear break-up time, Schirmer, corneal staining, meibomian gland, lid margin, and meibum. The specific search strategies are listed in Supplemental Digital Content (Supplementary Figure 1). No time restriction was applied to ensure the retrieval of broad published data. The last search was performed on 15 February, 2020. Limits were placed to retrieve only English-language and human studies. We then manually searched the references in the studies to identify any other potentially eligible studies. Duplicate studies were removed.

### Eligibility Criteria

Published peer-reviewed research articles were included in this review and meta-analysis. We could not conduct a meta-analysis of studies providing postoperative data for 1 week or 3 months after phacoemulsification because the lack of studies would have resulted in high heterogeneity and substantial publication bias. Therefore, only those studies with postoperative data for 1 month after phacoemulsification cataract surgery were included in this meta-analysis. Several other criteria were considered. (1) Some or all the study participants experienced uneventful phacoemulsification with or without intraocular lens implantation, with at least one of the following DED-related parameters recorded: dry eye questionnaire, BUT, CFS, Schirmer test without anesthesia (Schirmer I test). (2) Examinations were made both preoperatively and 1 month postoperatively. (3) All surgery was performed under topical anesthesia.

Articles were excluded if (1) the participants had a systemic disease that could interfere with tear film stability, including rheumatoid arthritis, gout, Stevens–Johnson syndrome, Sjögren disease, systemic lupus erythematosus, and multiple sclerosis, but patients with DM were not excluded from the study; (2) the patients had other eye diseases, including entropion, ectropion, uveitis, glaucoma, and severe fundus pathology; (3) the patients had a history of previous or concurrent use of topical or oral treatments (other than routine postoperative anti-inflammatory treatments) that could interfere with the outcome; (4) corneal sutures were made; (5) study of low quality, with was defined as existence of conspicuous inconsistencies in the article or a lack of demographic information; and (6) the outcomes were presented in an unextractable format (i.e., no corresponding standard deviation was provided with the outcome measurements or the data were presented in a qualitative or proportional manner).

### Data Collection

Data were extracted from the studies that fulfilled the inclusion and exclusion criteria, and Meta-analysis Of Observational Studies in Epidemiology (MOOSE) guidelines were used for abstracting data, assessing data quality and validity (24). Two reviewers (QL and XJZ) independently assessed the studies and extracted the data, and any inconsistencies were resolved by consensus. A standardized form was used to record the data on the authors of each study, the year of publication, the country of origin, the sample size, age, sex, size of incision, preoperative MGD status, DM, duration of follow-up, and outcome measures, including the baseline and postoperative parameters. Data were recorded as means ± standard deviations (SD). We interpolated the outcome measures from the figures if numerical values were not reported in the text and no raw data were received upon e-mail request. The results presented are based on eyes that had completed the follow-up.

### Risk of Bias Assessment

Because the studies included were not simply randomized clinical trials or comparative case–control studies, neither the Cochrane Collaboration tool nor the Ottawa–Newcastle Scale could be used (25). We were unable to find a validated tool to assess the risk of bias in single-arm case series. Sources of potential bias are addressed in the Discussion section.

### Statistical Analysis

All calculations were made with R 3.1.0 using the *metafor* package (26). For the dry eye questionnaire scores, the standardized mean change score using raw score standardization (SMCR) was calculated because there were discrepancies in the questionnaires used in each study and relatively large numerical disparities among the studies. The standardized mean change is defined as the mean difference between the posttest and pretest scores, which is standardized with the pretest standard deviation (26). Due to methodological homogeneity but heterogeneity in the score ranges, CFS was converted into ranges and calculated from the raw mean change (MC). To estimate the sampling variance of SMCR and MC, the pretest–posttest correlation (*r*_*c*_) was required. However, none of the studies reported *r*_*c*_. Because no raw data were received upon our request, we estimated *r*_*c*_ as (27, 28).

$$\begin{array}{l} r_{c}=\frac{SDbaseline^{2}+SDfinal^{2}-SDchange^{2}}{2\;\times \;SDbaseline\;\times \;SDfinal} \end{array}$$

*r*_*c*_ = pretest–posttest correlation; *SDbaseline* = standard deviation (SD) of preoperative test value; *SDfinal* = SD of postoperative test value; *SDchange* = SD of the change in test value.

Only two studies (29, 30) reported *SDchange*, so *r*_*c*_ was estimated as the average of calculated *r*_*c*_ for those that did not report it. The effect size (SMCR and MC) was calculated with the *escalc* function in the *metafor* package for each outcome variable. We performed the meta-analysis using data that were collected 1 month postoperatively and compared with the baseline outcome measures.

The heterogeneity of the studies included was evaluated with the χ2 test and by examining the I^2^ value. If there was significant statistical heterogeneity among the studies, a random-effects model was used to pool the data; otherwise, a fixed-effects model was used (1, 31). A sensitivity analysis was also performed by excluding one study at a time to evaluate the reliability of each study (32). Publication bias was assessed with funnel plots, Begg's test (rank correlation method) and Egger's test (linear regression method) (33). Meta-regression was used to examine the differences between studies in terms of such possibly influential factors as preoperative MGD status, DM status (21, 22), the corresponding preoperative DED-related parameters, the size of incision (9, 23), and the country of origin. Subgroup analyses were performed based on the following meta-regression results: preoperative MGD status (cataract patients with various degrees of preoperative MGD and studies with unspecified preoperative MGD status); DM status (a specified proportion of cataract patients diagnosed with DM, cataract patients without DM or systemic disease, and studies with unspecified DM status). *P* < 0.05 was considered statistically significant for all two-sided tests.

## Results

**Figure 1:**
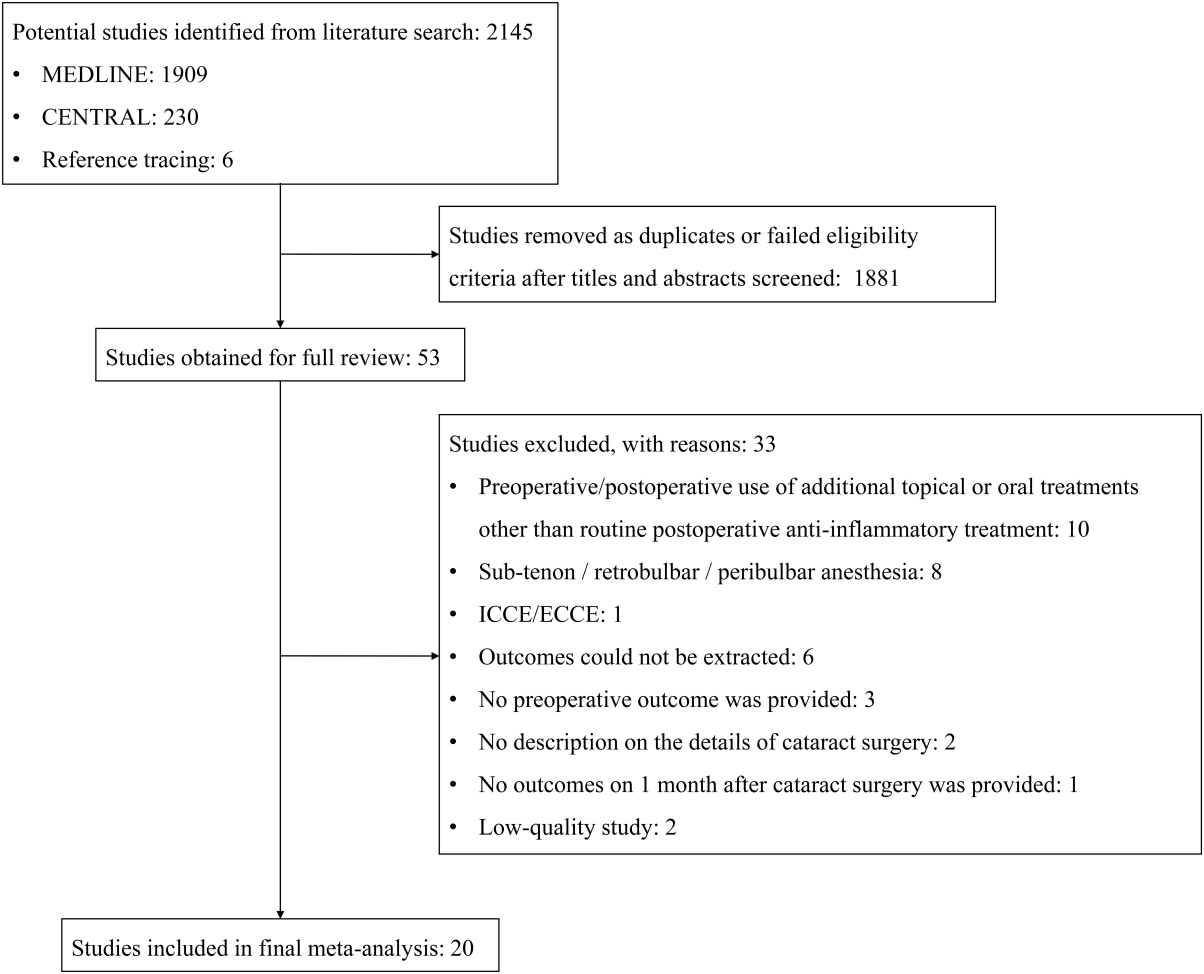
Flow diagram of the selection process for studies included in the meta-analysis. CENTRAL, Cochrane Central Register of Controlled Trials; ICCE, intracapsular cataract extraction; ECCE, extracapsular cataract extraction.

A total of 2,145 articles were identified with the initial database search, 20 of which were finally included, involving a total of 2,247 eyes (Figure 1). The basic characteristics of the eligible studies and methods used to report various ocular surface parameters are described in Tables 1, 2, respectively. All studies included used similar surgical procedure and postoperative management (Table 3).

**Table 1:** Characteristics of the included studies.

**Study**	**Year of Publication**	**Country**	**Sample size (number of eyes)**	**Age (Mean ± SD)**	**Gender (M/F)**	**Incision (mm)**	**MGD status**	**DM status**	**Follow-up period**
A. El Ameen (34)	2018	France	30	75.7 ± 8.2	18/12	1.8	Not mentioned^*^.	Not mentioned	1 m, 3 m
Ana Gonzalez-Mesa (35)	2016	Spain	52	70.7 ± 7.9	28/24	2.75	Not mentioned.	Not mentioned	1 m, 3 m
Dewang Shao (11)	2018	China	150	69.1 ± 12.6	62/88	2.2	Not mentioned.	Without DM	1 w, 1 m, 3 m
Do Yeh Yoon (19)	2019	Korea	11	50–75	N/A	2.2	Not mentioned.	Without systemic disease	1 w, 1 m
Donghong Jiang (36)	2016	China	568	65.3 ± 12.0	258/310	3.4–3.8	Not mentioned.	174 (27%) DM474 (73%) without DM	1 w, 1 m, 3 m
Hoseok Moon (37)	2014	Korea	29	63.2 ± 14.0	12/17	2.75	Not mentioned.	Not mentioned	1 w, 1 m
Hun Lee (38)	2016	Korea	33	66.7 ± 9.0	10/23	NA	Not mentioned.	Not mentioned	1 m, 3 m
Ji Won Jung (39)	2016	Korea	50	67.0 ± 10.1	11/39	2.8	35 eyes (70%) with No MGD or grade 1 MGD; 15 eyes (30%) with Grade 2 or higher MGD.	Not mentioned	1 m
Jin Sun Kim (13)	2016	Korea	43	65.0 ± 13.8	30/13	NA	Mild to moderate MGD.	Without systemic disease	1 m, 3 m
Ke Yao (29)	2015	China	90	69.0 ± 7.1	31/59	NA	Not mentioned.	26 (29%) DM64 (71%) without DM	1 w, 1 m
Kensaku Miyake (40)	2017	Japan	433	71.9 ± 7.5	199/234	2.32 ± 0.14	Not mentioned.	Not mentioned	1 m
Kyung Eun Han (12)	2014	Korea	58	68.3 ± 11.7	21/27	3.2	No to moderate obstructive MGD.	Not mentioned	1 m, 3 m
MA Sa'nchez (41)	2010	Spain	21	72.4 ± 6.4	12/9	NA	Not mentioned.	Without systemic disease	1 m
Maierhaba Yusufu (42)	2017	China	30	64.7 ± 11.4	17/13	2.6	Not mentioned.	14 (47%) DM16 (53%) without DM	1 w, 1 m
Maria Garcia Zamora (17)	2020	Spain	55	75.8 ± 7.3	27/28	2.75	Not mentioned.	Not mentioned	1 w, 1 m
Rita Mencucci (43)	2015	Italy	136	70.3	56/80	2.2	Not mentioned.	Not mentioned	1 w, 5 w
Taehoon Oh (16)	2012	Korea	48	62.0 ± 9.7	10/20	2.8	Not mentioned.	Not mentioned	1 m, 3 m
Yinhui Yu (30)	2015	China	64	71.8 ± 10.1	27/37	NA	Not mentioned.	Without DM	1 w, 1 m
Young Joon Choi (18)	2018	Korea	116	66.3 ± 10.7	54/62	2.8	Not mentioned^*^.	Not mentioned	1 m, 3 m
Yuli Park (9)	2016	Korea	34	65.8 ± 6.1	6/12	2.85	Not mentioned.	Not mentioned	1 d, 1 m, 2 m

*MGD, meibomian gland dysfunction; DED, dry eye disease; DM, diabetic mellitus; w, week(s); m, month(s); N/A, data not available*.

* *Though A. El Ameen et al. (34) and Young Joon Choi et al. (18) mentioned a preoperative meibomian gland loss, their subjective grading scale based on meibomian gland structure area tends to overestimate the dropout (44). Considering their lack of explicit mentioning of preoperative MGD status, these two studies were categorized as “not mentioned” when referring to preoperative MGD status*.

**Table 2:** Methods that the included studies used to report ocular surface variables.

**Study**	**Year of Publication**	**Questionnaires (range)**	**BUT**	**CFS (range)**	**Schirmer I test**
A. El Ameen (34)	2018	OSDI item 4&5 eliminated (0–100)	NIKBUT Keratograph 5M (OCULUS, Germany)	Oxford staining scores (0–5)	N/A
Ana Gonzalez-Mesa (35)	2016	OSDI (0–100)	N/A	Oxford staining scores (0–5)	N/A
Dewang Shao (11)	2018	OSDI (0–100)	NIfBUT corneal topographer (Oculus Optikgerate GmbH, Germany)	Four quadrants staining score (0–12)	without anesthesia
Do Yeh Yoon (19)	2019	OSDI (0–48)	BUT	Numerical point scale (0–3)	N/A
Donghong Jiang (36)	2016	OSDI (0–48)	BUT	NEI-recommended guidelines staining score (0–15)	without anesthesia
Hoseok Moon (37)	2014	OSDI (0–100)	BUT	N/A	N/A
Hun Lee (38)	2016	OSDI (0–48)	BUT	Oxford staining scores (0–5)	without anesthesia
Ji Won Jung (39)	2016	OSDI (0–100)	BUT	Oxford staining scores (0–5)	without anesthesia
Jin Sun Kim (13)	2016	OSDI (0–100)	BUT	Oxford staining scores (0–5)	without anesthesia
Ke Yao (29)	2015	OSDI (0–48)	BUT	4-point scale ×3 region (0–9)	with anesthesia
Kensaku Miyake (40)	2017	subjective symptom (0–36)	BUT	Corneal and conjunctival staining (0–9)	without anesthesia
Kyung Eun Han (12)	2014	Ocular symptom score (0–14)	BUT	N/A	without anesthesia
MA Sa'nchez (41)	2010	OSDI (0–100)	BUT	Oxford staining scores (0–5)	with anesthesia
Maierhaba Yusufu (42)	2017	OSDI (0–100)	NIKBUT Keratograph 5M (OCULUS, Germany)	Four-point scale (0–3)	without anesthesia
Maria Garcia Zamora (17)	2020	OSDI (0–100)	BUT	NEI-recommended guidelines staining score (0–15)	without anesthesia
Rita Mencucci (43)	2015	N/A	BUT	N/A	N/A
Taehoon Oh (16)	2012	OSDI item 4&5 eliminated (0–4)	BUT	N/A	without anesthesia
Yinhui Yu (30)	2015	OSDI (0–100)	NIfBUT Corneal topographer (Oculus Optikgerate GmbH, Germany)	Oxford staining scores (0–5)	without anesthesia
Young Joon Choi (18)	2018	OSDI (0–100)	BUT	Oxford staining scores (0–5)	without anesthesia
Yuli Park (9)	2016	OSDI item 4&5 eliminated (0–4)	BUT	NEI-recommended guidelines staining score (0–15)	without anesthesia

*BUT, tear break-up time; CFS, corneal fluorescein staining; OSDI, Ocular Surface Disease Index; N/A, data not available; NIKBUT, non-invasive keratographic tear break-up time; NIfBUT, non-invasive first tear break-up time; NEI, The National Eye Institute/Industry*.

**Table 3:** Surgical methods and postoperative management.

**Study**	**Year of Publication**	**Anesthesia**	**Surgical methods**	**Postoperative management (medication; frequency; duration)**
A. El Ameen (34)	2018	Topical	Phacoemulsification + IOL implantation	0.5% tropicamide eye drops; three times/day; 5 days 1.5% azithromycin eye drops; two times/day; 3 days 0.1% dexamethasone phosphate, 0.1% indomethacin eye drops; three times/day; 1 month
Ana Gonzalez-Mesa (35)	2016	Topical	Phacoemulsification + IOL implantation	Tobradex (tobramycin, dexamethasone) eye drops; frequency not mentioned, 4 weeks
Dewang Shao (11)	2018	Topical	Phacoemulsification + IOL implantation	Tobramycin, dexamethasone, pranoprofen eye drops; four times/day in the 1st week, decreased progressively by 1 day every week; 4 weeks
Do Yeh Yoon (19)	2019	Topical	Phacoemulsification + IOL implantation	Not mentioned
Donghong Jiang (36)	2016	Topical	Phacoemulsification + IOL implantation	0.3% tobramycin/0.1% dexamethasone, 0.1% pranoprofen eye drops; three times/day 4 weeks
Hoseok Moon (37)	2014	Topical	Phacoemulsification + IOL implantation	Not mentioned
Hun Lee (38)	2016	Topical	Phacoemulsification + IOL implantation	Gatifloxacin, 1% prednisolone acetate eye drops; four times/day; 1 month
Ji Won Jung (39)	2016	Topical	Phacoemulsification + IOL implantation	Levofloxacin, 1% prednisolone acetate eye drops; four times/day; 4 weeks
Jin Sun Kim (13)	2016	Topical	Phacoemulsification + IOL implantation	0.5% levofloxacin, 1% prednisolone acetate eye drops; four times/day; 4 weeks
Ke Yao (29)	2015	Topical	Phacoemulsification + IOL implantation	Conventional post-surgical therapy; frequency not mentioned 1 month
Kensaku Miyake (40)	2017	413 Topical+ 2 retrobulbar	Phacoemulsification + IOL implantation	1.5% levofloxacin, 0.1% fluorometholone eye drops; frequency not mentioned; 4 weeks 0.1% diclofenac sodium eye drops; frequency not mentioned; 8 weeks
Kyung Eun Han (12)	2014	Topical	Phacoemulsification + IOL implantation	0.5% levofloxacin, 1% prednisolone acetate eye drops; four times/day; 4 weeks
MA Sa'nchez (41)	2010	Topical	Phacoemulsification + IOL implantation	Tobramycin, dexamethasone eye drops; four times/day for the 1st week, tapering the dose for a further 3 weeks; 4 weeks
Maierhaba Yusufu (42)	2017	Topical	Phacoemulsification + IOL implantation	0.5% levofloxacin, 1% prednisolone acetate eye drops; three times/day; 2 weeks0.1% pranoprofen eye drops; four times/day in the 1st week, two times/day for the 2nd week, one time/day for the 3rd week; 3 weeks
Maria Garcia Zamora (17)	2020	Topical	Phacoemulsification + IOL implantation	Tobramycin, dexamethasone eye drops; four times/day for the 1st week, tapering the dose for a further 3 weeks; 4 weeks
Rita Mencucci (43)	2015	Not mentioned	Phacoemulsification + IOL implantation	0.3% tobramycin, 0.1% dexamethasone acetate eye drops; four times/day in the 1st week, decreased progressively by 1 day every week; 4 weeks
Taehoon Oh (16)	2012	Topical	Phacoemulsification + IOL implantation	0.3% gatifloxacin eye drops; four times/day, 1 month1% prednisolone acetate eye drops; four times/day;
Yinhui Yu (30)	2015	Topical	Phacoemulsification + IOL implantation	Dexamethasone-tobramycin eye drops; four times/day, 2 weeks. Pranoprofen eye drops; four times/day; 1 month
Young Joon Choi (18)	2018	Topical	Phacoemulsification + IOL implantation	0.5% levofloxacin, 1% prednisolone acetate; four times/day; 4 weeks
Yuli Park (9)	2016	Not mentioned	Phacoemulsification + IOL implantation	0.5% moxifloxacin, 1% prednisolone acetate eye drops; four times/day; 1 month

Of the studies included, two were randomized controlled clinical trials (11, 43), nine were prospective non-randomized comparative cohort studies (9, 19, 29, 30, 36, 37, 39, 41, 42), eight were prospective interventional self-controlled studies (12, 13, 16–18, 34, 35, 40), and one was a retrospective comparative observational case series (38).

### Publication Bias

Funnel plots were used to visually identify a potential publication bias, no obvious asymmetry was observed, and the outliers could partly be explained by the high heterogeneity of the studies (Supplementary Figure 2). The corrected publication bias using the “trim and fill” method remained unchanged. Neither Begg's test nor Egger's test detected evidence of publication bias in the dry eye questionnaires (*P* = 0.89 and 0.42, respectively), BUT (*P* = 0.24 and 0.45, respectively), CFS (*P* = 0.96 and 0.37, respectively), or the Schirmer I test (*P* = 0.68 and 0.83, respectively).

### Interaction Test Analysis Using Meta-Regression

According to tests for interaction using meta-regression (Table 4), the preoperative MGD status was associated with the scores for subjective symptoms, BUT and CFS, while DM status influenced the results of the Schirmer I test. The scores for subjective symptoms and CFS were associated with their corresponding preoperative test values. Therefore, we performed a further subgroup analysis according to the meta-regression results and stratified the studies by preoperative MGD in an analysis of subjective symptoms, BUT and the CFS score. The results for the Schirmer I test was stratified according to DM status.

**Table 4:** Interaction test analysis using meta-regression.

	**Preoperative MGD status**	**DM status**	**Preoperative test value**	**Size of incision**	**Nation**
Questionnaires	0.04^a^	0.72	0.0001^a^ (β = −0.07)	0.26 (β = 0.70)	0.10
BUT	<0.0001^a^	0.16	0.56 (β = −0.06)	0.29 (β = −0.56)	0.64
CFS	0.01^a^	0.86	0.03^a^ (β = −0.19)	0.82 (β = −0.06)	0.13
Schirmer I test	0.51	<0.0001^a^	0.85 (β = 0.03)	0.53 (β = 0.47)	0.77

*MGD, meibomian gland dysfunction; DED, dry eye disease; DM, diabetic mellitus; BUT, tear break-up time; CFS, corneal fluorescein staining; NA, not available*.

a *Statistically significant (P < 0.05)*.

### DED-Related Parameters

#### Dry Eye Questionnaires

**Figure 2:**
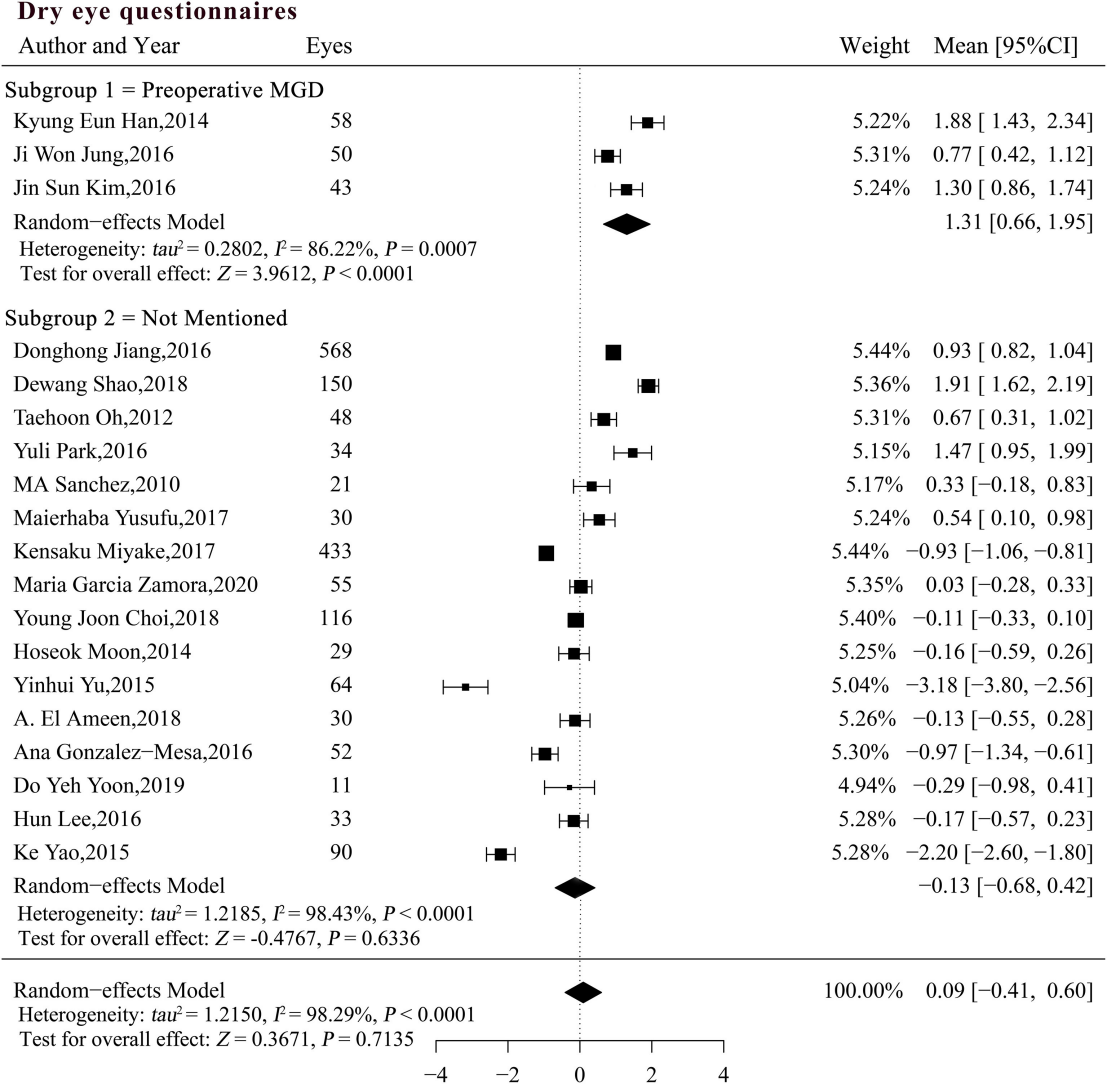
Forest plots of change in dry eye questionnaires after phacoemulsification cataract surgery. CI, confidence interval; MGD, meibomian gland dysfunction; DED, dry eye disease.

According to the tests for interaction, the preoperative MGD status (*P* = 0.04) and pre-existing subjective symptoms (β = −0.07, *P* = 0.0001) may explain the study heterogeneity (Table 4). According to the meta-analysis, patients with pre-existing MGD complained significantly more strongly of dry eye after phacoemulsification cataract surgery (*SMCR*_*subgroup*1_ = 1.31, 95% confidence interval (CI) [0.66, 1.95], *P* < 0.0001), which differed significantly from the results in patients with unspecified MGD status [*P* = 0.001; Figure 2 (data were listed by preoperative questionnaire score in ascending order)]. The heterogeneity among subgroups was analyzed with a random-effects model, and the results remained robust in the sensitivity analysis (Supplementary Table 1).

#### Tear BUT

**Figure 3:**
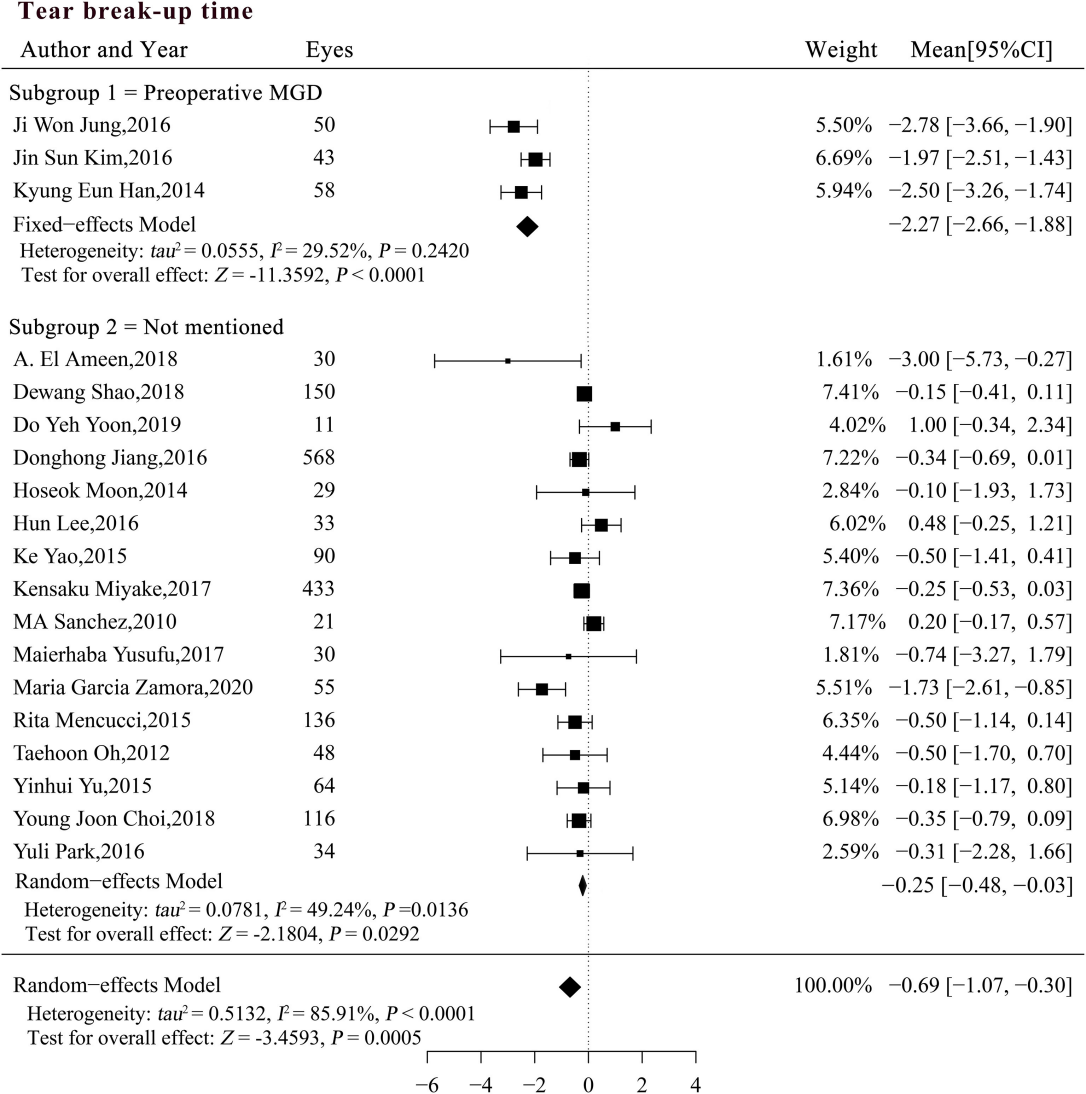
Forest plots of change in tear break-up time after phacoemulsification cataract surgery. CI, confidence interval; MGD, meibomian gland dysfunction; DED, dry eye disease.

The preoperative MGD status may explain the heterogeneity of the studies when BUT was pooled for analysis (*P* < 0.0001; Table 4). A subgroup analysis was performed based on the preoperative MGD status and significant differences were detected among the subgroups (Figure 3). The reduction in BUT was numerically and statistically significant in both subgroups (*MC*_*subgroup*1_ = −2.27, 95% CI [−2.66, −1.88], *P* < 0.0001; *MC*_*subgroup*2_ = −0.25, 95% CI [−0.48, −0.03], *P* = 0.0292), but the patients with pre-existing MGD showed a more significant reduction in BUT compared with those with unspecified MGD status (*P* < 0.0001). A sensitivity analysis showed that the significant difference between two subgroups was robust, whereas the result in patients with unspecified MGD was not very stable after exclusion of studies with large sample size (Supplementary Table 2).

#### Corneal Fluorescein Staining

**Figure 4:**
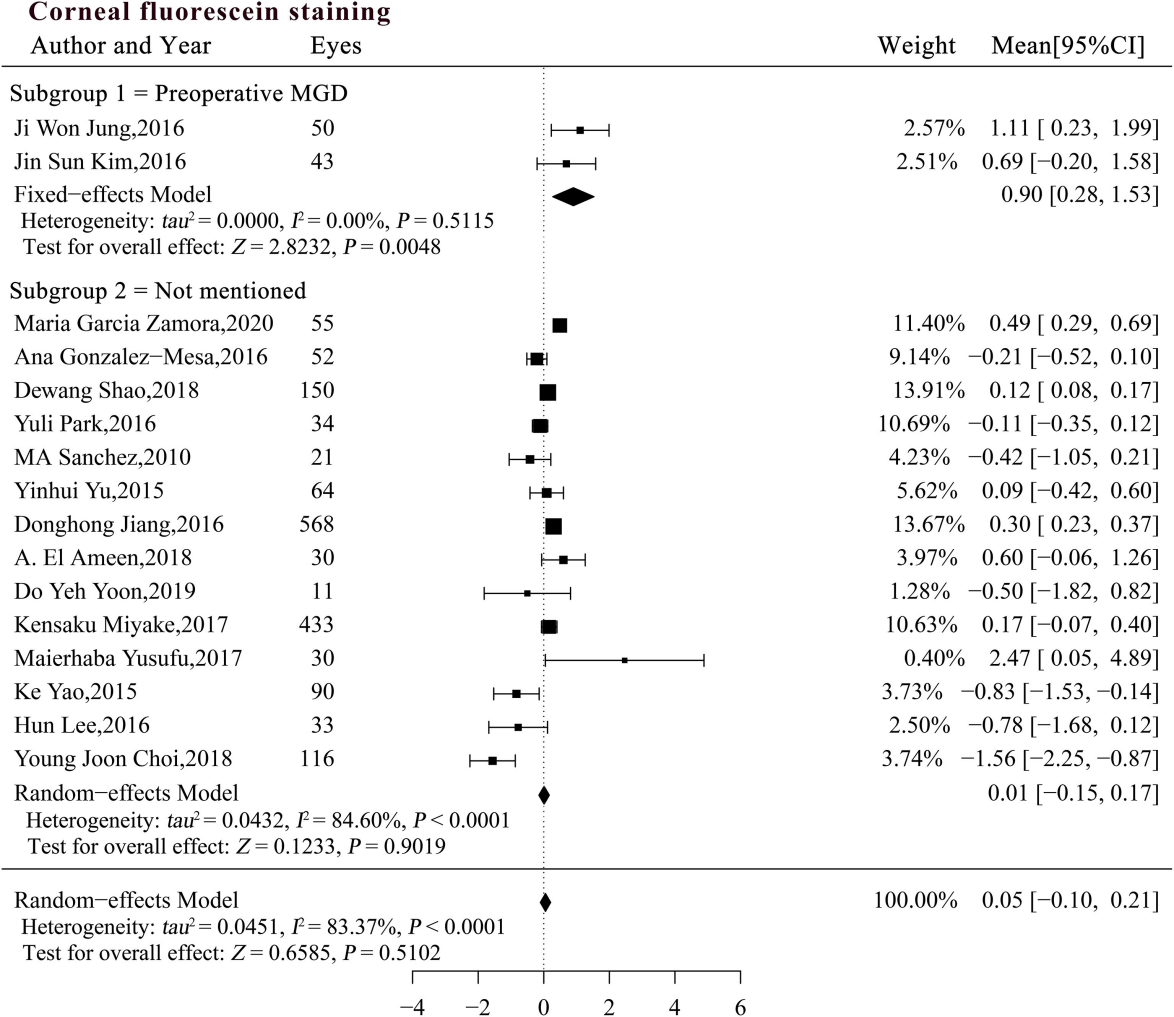
Forest plots of change in corneal fluorescein staining after phacoemulsification cataract surgery. CI, confidence interval.

The interaction test indicated that both MGD status (*P* = 0.01) and pre-existing CFS score (β = −0.19, *P* = 0.03) may explain the heterogeneity in the studies (Table 4). The pooled MC for CFS was significantly increased in patients with pre-existing MGD (*MC*_*subgroup*1_ = 0.90, 95% CI [0.28, 1.53], *P* = 0.0048), but did not change significantly in patients with an unspecified MGD status [*MC*_*subgroup*2_ = 0.01, 95% CI [-0.15, 0.17], *P* = 0.9019; Figure 4 (data were listed by preoperative CFS in ascending order)]. Patients with pre-existing MGD had significantly greater deterioration of the corneal surface than patients with an unspecified preoperative MGD status (*P* = 0.005). Sensitivity analysis indicated that the combined result in subgroup 1 was not robust enough due to limited sample size, while exclusion of a single study in subgroups 2 did not materially alter the pooled results (Supplementary Table 3).

#### Schirmer I Test

**Figure 5:**
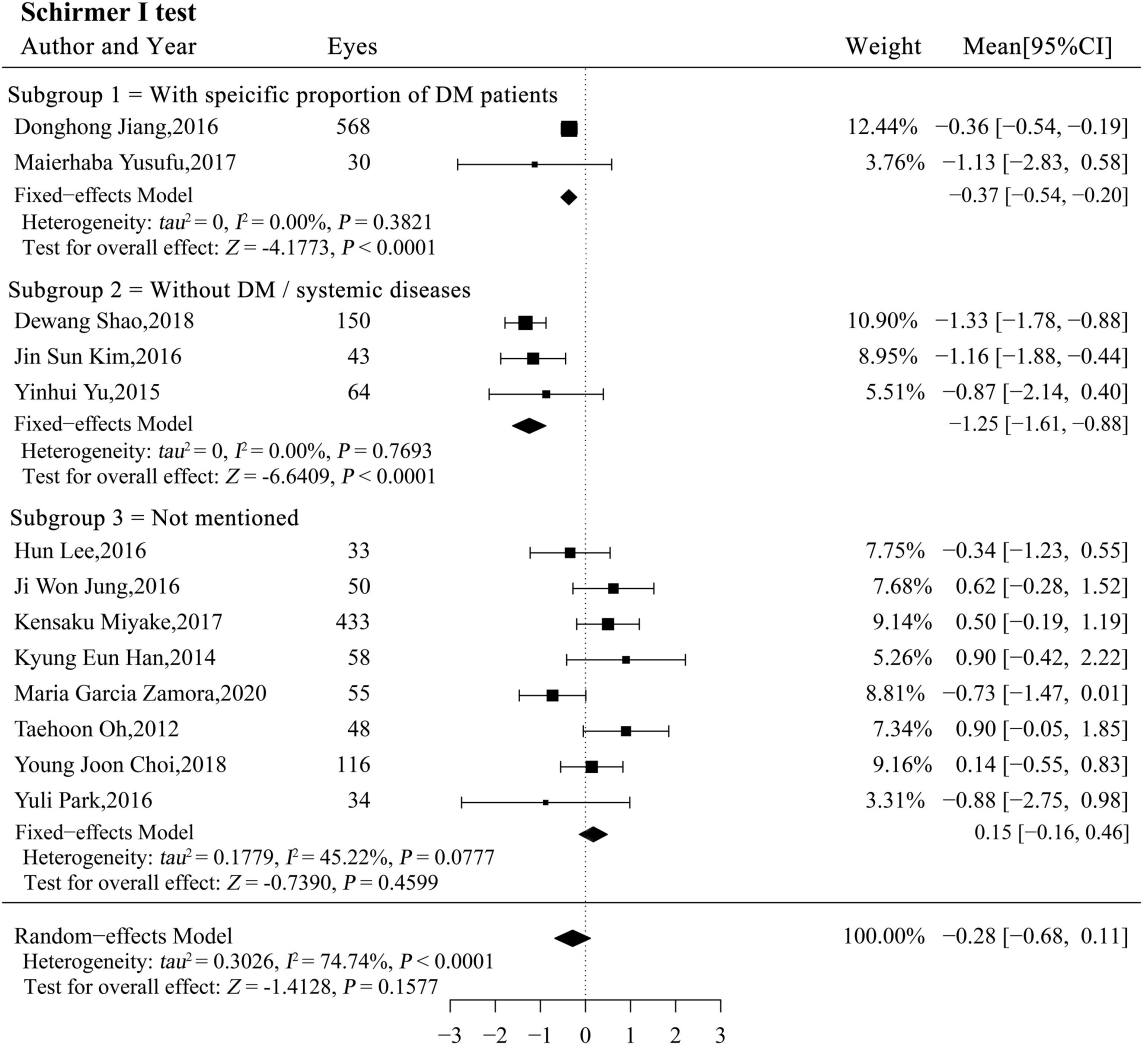
Forest plots of change in the Schirmer I test after phacoemulsification cataract surgery. CI, confidence interval; DM, diabetes mellitus.

The result of the Schirmer I test showed that the DM status potentially altered tear secretion and may be a source of heterogeneity (*P* < 0.0001; Table 4). We stratified the studies by patient DM status and performed a subgroup analysis. Total tear secretion decreased significantly after phacoemulsification cataract surgery in studies with a specific proportion of DM patients and in studies of patients without DM (*MC*_*subgroup*1_ = −0.37, 95% CI [−0.54, −0.20], *P* < 0.0001; *MC*_*subgroup*2_= −1.25, 95% CI [−1.62, −0.88], *P* < 0.0001; Figure 5). However, no significant difference in tear secretion was observed in patients with an unspecified DM status (*MC*_*subgroup*3_ = 0.15, 95% CI [−0.16, 0.46], *P* = 0.3345). Significantly more decrease in tearing was observed in patients without DM compared with two other subgroups (both *P* < 0.001), and studies expounding specific proportion of DM patients also showed more decrease in tearing compared with those with unspecified DM status (*P* = 0.03). There was no significant heterogeneity within all three subgroups. According to sensitivity analysis, the significant difference between subgroups without DM and with unspecified DM status remained robust. However, the difference between subgroups with a specific proportion of DM patients and with unspecified DM was not stable and could be substantially altered by exclusions of a single study (Supplementary Table 4).

## Discussion

This systematic review and meta-analysis summarizes the current knowledge on ocular surface changes that occur in patients after phacoemulsification cataract surgery. A total of 20 trials were included in the meta-analysis, involving 2,247 eyes. Generally, the cataract patients did not suffer from a deteriorated ocular surface in terms of DED-related parameters after surgery. This study shows that cataract patients with pre-existing MGD more frequently suffered irritating symptoms, reduced tear film stability (evident as shortened BUT), and damage to the corneal surface than those without pre-existing MGD. Cataract patients without DM were more likely to experience a reduction in postoperative tearing.

### Pre-existing MGD

The significant deterioration of subjective symptoms, BUT, and CFS was observed after phacoemulsification cataract surgery in patients with pre-existing MGD. While in studies that did not mention MGD status, these DED-related parameters generally remained unchanged after phacoemulsification cataract surgery.

Dry eye symptoms were exacerbated more severely in patients with pre-existing MGD. These irritating ocular symptoms are largely determined by the tear film stability, measured as BUT, and can be significantly aggravated even with a normal Schirmer I test result and no epithelial damage (45). Notably, significant heterogeneity was observed when the subjective symptoms were pooled for analysis, even after the subgroup analysis. Discrepancies arose from the variability in measurements and intrinsic individual differences, and differences in the prevalence and severity of preoperative MGD may also explain this heterogeneity. The severity of symptoms is reported to correlate poorly with clinical signs, and many individuals showed conflicting symptoms and signs (44, 46, 47). Previous studies have proposed that any effect of a reduction of ≤ 4.1 in the Ocular Surface Disease Index (range: 0–48) may be subclinical (48). However, in this meta-analysis, SMCR was used to accommodate the methodological diversity, so no quantitative conclusion could be drawn.

The deterioration in tear film stability (as indicated by BUT) after phacoemulsification cataract surgery may be largely attributable to pre-existing MGD. Eyes with preoperative MGD were already in a subclinical inflammatory microenvironment. Aggravating factors associated with phacoemulsification cataract surgery could exacerbate this inflammation, leading to eyelid congestion and increased swelling, which was not compensated for as well in the preoperative MGD group as in the non-MGD groups (7, 49, 50). We believe that intraoperative irritation, postoperative preservative-containing eye drops, the release of inflammatory mediators, and lid dysfunction resulting from the use of a lid speculum could all be factors that aggravate MGD (9, 10, 51). Consequently, there is increased intraglandular pressure and more difficult meibum discharge. The deposition of meibum could promote bacterial growth, producing detrimental mediators and leading to adverse changes in meibum production. Therefore, BUT, which is largely determined by meibum and reflects tear film stability, showed significant deterioration in patients with pre-existing MGD. This reduction in BUT was smaller in the general population, and was not significant enough according to sensitivity analysis. Therefore, we speculate that such discrepancy could be due to worsened Meibomian gland function after the surgery in patients with pre-existing MGD (7, 34).

The CFS score had a significant postoperative increase probably as a result of direct intraoperative physical damage and postoperative eye drop use. Previous studies have considered that postoperative CFS correlates positively with inflammatory cytokine levels and that higher levels of ocular surface inflammation compromises corneal health (9, 52). Patients with pre-existing MGD, even when receiving routine postoperative anti-inflammatory treatment, had higher levels of inflammatory mediators than patients without pre-existing MGD (9). Therefore, patients with MGD were more likely to experience slower corneal recovery, whereas the CFS score remained unchanged in patients with no or a lower level of postoperative MGD.

Moreover, changes in the CFS score correlated negatively with the preoperative test value. Because the CFS score is semiquantitative and phacoemulsification cataract surgery can harm the ocular surface, an increase in the CFS score from grade 0 (no staining) to grade 1 (superficial stippling and micropunctate staining) may be more easily achieved than an increase from grade 1 to 2 (macropunctate staining with some coalescent areas) (7). The postoperative use of eye drops and proper postoperative management, including the avoidance of eye rubbing and adequate blinking, might improve the epithelial damage in eyes with high preoperative CFS scores (18). These factors may explain the negative correlation between the preoperative CFS score and the change in CFS after phacoemulsification cataract surgery.

### DM

According to our meta-analysis, the total changes in tear secretion detected with the Schirmer I test were significantly affected by DM. DM has long been identified as a risk factor for DED (53), with asymptomatic and symptomatic dry eye affecting up to 54% of diabetes patients (54). Studies included in our meta-analysis all conducted Schirmer I test without anesthesia, whose result was more associated with reflex tearing. Reflex tearing is closely related to corneal sensitivity. The cornea has the highest density of sensory nerve endings in the human body, and an incision made during phacoemulsification cataract surgery can reduce corneal sensitivity and tear secretion (23, 55, 56), and larger incisions together with its subsequent inflammation could reduce corneal sensitivity more than smaller incisions (9, 23). Interestingly, our results indicated that patients without diabetes were more susceptible to reduced tearing after phacoemulsification cataract surgery. In DM patients, both microvascular damage and peripheral neuropathy could reduce corneal sensitivity and reduce reflex tearing (52, 57, 58), which reduced the effects of corneal nerve transection in DM patients during incision. However, reflex tearing was also significantly reduced in the subgroup of studies that reported the proportions of DM patients, which was mainly influenced by the results of Jiang et al. (36). This may be due to the large incisions made by Jiang et al. (36), which were 3.4–3.8 mm (other studies reported incision sizes of 1.8–3.2 mm). Besides, it was indicated that the duration of DM and its control status may also affect the incidence of DED (21), yet no previous study has combined these DED-influencing factors with cataract surgery. We expect further studies to demonstrate the relationship between a more comprehensive DM status and postoperative dry eye.

### Study Limitations

Several other limitations were encountered in reviewing this literature. The first was the lack of a validated tool to assess quality of publications, which generated a risk-of-bias evaluation. There was inevitable lack of allocation concealment or blinding of patients and researchers, thus leading to selection bias and performance bias. Besides, only five studies in total reported loss of follow-up but without detailed explication and this would be a risk for attrition bias. Results were observational, obtained based on self-control, thus indication for treatment biases might exist (59). We tried other approaches to evaluate manuscript quality. The Oxford Center for Evidence-Based Medicine's Level of Evidence system (25) would put the studies included into level 4 (“case series and poor-quality cohort or case–control studies”) and the Grades of Recommendation Assessment, Development and Evaluation system (60) would categorize them as a “low level of evidence.” Second, no individual-level data became available, even upon request, so it was impossible to adjust for various potentially confounding attributes, and thus the correlation coefficients were estimates. Third, significant heterogeneity was observed when the data were pooled for analysis, even in the subgroup analyses. This limitation arises from a lack of universal grading of MGD, different proportion of preoperative MGD patients as well as the inherent design of a systematic review, which relies on published findings that could be substantially affected by the clinical tests or operations used or by the way the authors chose to present their findings.

### Clinical Significance

Cataract surgery today is not only a technique to improve visual acuity, but also a route to a better quality of life. Therefore, the elimination of complications and unpleasant sensations, arising both intraoperatively and postoperatively, has become a new challenge for ophthalmologists. The causes of dry eye after phacoemulsification cataract surgery are multifactorial, and studies of the pathogenesis of DED have made the condition largely predictable. The perioperative management of manageable symptoms, such as pre-existing MGD, can be effective and even the optimal method for alleviating DED after phacoemulsification cataract surgery (7). In patients with long-term systemic diseases, such as DM, a preoperative explanation of the possible postsurgical DED could be helpful because although the symptoms and signs of dry eye can lead to patient dissatisfaction and concern about postoperative complications, patients are more accepting if they were informed of possible symptoms before surgery. Therefore, it is important to understand DED after phacoemulsification cataract surgery properly, and clinicians should provide both preoperative treatment and an explanation of postoperative dry eye symptoms.

The findings of this systematic review and meta-analysis summarize the current knowledge of ocular surface characteristics after phacoemulsification cataract surgery. This meta-analysis examined the comprehensive parameters affecting DED and compared the 1-month-postoperative values with the corresponding preoperative values. According to our meta-analysis, phacoemulsification cataract surgery does not induce or exacerbate DED in the general population except for slightly reduced tear film stability. Whereas, cataract patients with pre-existing MGD are more likely to suffer irritation symptoms, more disrupted tear film stability, and a damaged corneal surface. Non-DM cataract patients are more susceptible to corneal nerve transection caused by incisions and display reduced reflex tearing after surgery compared with patients with DM. Extending our knowledge of dry eye after phacoemulsification cataract surgery will allow surgeons to pinpoint at-risk patients and provide the explanations and precautions required for better overall outcomes in clinical practice.

## Data Availability Statement

The original contributions generated for this study are included in the article/Supplementary Material, further inquiries can be directed to the corresponding author/s.

## Author Contributions

QL: conceptualization, methodology, software, roles/writing - original draft, and writing - review & editing. YL: conceptualization, funding acquisition, project administration, supervision, and validation. XZ: conceptualization, funding acquisition, project administration, writing - review & editing, supervision, and validation. All authors contributed to the article and approved the submitted version.

## Conflict of Interest

The authors declare that the research was conducted in the absence of any commercial or financial relationships that could be construed as a potential conflict of interest.

## Supplementary Material

Supplementary Figure 1 Search strategies.

Supplementary Figure 2 Funnel plots for **(A)** dry eye questionnaires against standard error (SE); **(B)** dry eye questionnaires against sample size; **(C)** tear break-up time against SE; **(D)** tear break-up time against sample size; **(E)** corneal fluorescein staining score against SE **(F)** corneal fluorescein staining score against sample size; **(G)** Schirmer I test against SE; **(H)** Schirmer I test against sample size.

Supplementary Table 1 Sensitivity analyses on dry eye questionnaires by excluding one study at a time.

Supplementary Table 2 Sensitivity analyses on tear break-up time by excluding one study at a time.

Supplementary Table 3 Sensitivity analyses on corneal fluorescein staining by excluding one study at a time.

Supplementary Table 4 Sensitivity analyses on Schirmer I test by excluding one study at a time.
