# Supplementary material for: Dry Eye and Phacoemulsification Cataract Surgery: A Systematic Review and Meta-Analysis
Source: Front Med (Lausanne). 2021 Jul 8;8:649030. doi: 10.3389/fmed.2021.649030 (PMC8295542; doi:10.3389/fmed.2021.649030)
Supplement: Supplementary file 1 [file Table_1.DOC]

**MOOSE Checklist**

**Dry eye and cataract surgery: A systemic review and meta-analysis**

Qiang Lu, MD^1^, Yi Lu, MD^1^, Xiangjia Zhu, MD^1*^

Affiliations:

^1^ Eye Institute and Department of Ophthalmology, Eye & ENT Hospital, Fudan University, Shanghai 2000031, China; NHC Key Laboratory of Myopia (Fudan University); Key Laboratory of Myopia, Chinese Academy of Medical Sciences, Shanghai, 200031, China; Shanghai Key Laboratory of Visual Impariment and Restoration, Shanghai 200031, China.

*Corresponding author:

Xiangjia Zhu

Department of Ophthalmology, Eye and Ear, Nose, and Throat Hospital of Fudan University, 83 Fenyang Road, Shanghai 200031, China;

Tel: +86 21 64377134-407;

Fax: +86 21 64318258;

E-mail: [zhuxiangjia1982@126.com](mailto:zhuxiangjia1982@126.com)

| **Criteria** | | **Brief description of how the criteria were handled in the meta-analysis** |
| --- | --- | --- |
| **Reporting of background should include** | | |
| √ | Problem definition | Dry eye is one of the commonest complaints reported in ophthalmology clinics, currently accounting for 17%–25% of outpatient visits. The prevalence of dry eye disease (DED) ranges from 6% to 34%.  Although there are many potentially DED-inducing factors both intraoperatively and postoperatively, whether cataract surgery is a risk factor for DED remains controversial. |
| √ | Hypothesis statement | Cataract surgery aggravate dry eye disease. |
| √ | Description of study outcomes | DED-related parameters: subjective questionnaires, tear break-up time, corneal fluorescein staining, Schirmer test. |
| √ | Type of exposure or intervention used | Cataract surgery. |
| √ | Type of study designs used | Of the studies included, two were randomized controlled clinical trials, nine were prospective nonrandomized comparative cohort studies, eight were prospective interventional self-controlled studies, and one was a retrospective comparative observational case series |
| √ | Study population | We placed no restriction. |
| **Reporting of search strategy should include** | | |
| √ | Qualifications of searchers | The credentials of the two investigators CJ and MM are indicated in the author list. |
| √ | Search strategy, including time period included in the synthesis and keywords | PubMed: before February 2020  Cochrane Central Register of Controlled Trials (CENTRAL): before February 2020  See Figure 1 in the article |
| √ | Databases and registries searched | PubMed and CENTRAL |
| √ | Search software used, name and version, including special features | We did not employ a search software. NoteExpress was used to merge retrieved citations and eliminate duplications. |
| √ | Use of hand searching | We hand-searched bibliographies of retrieved papers for additional references, |
| √ | List of citations located and those excluded, including justifications | Details of the literature search process are outlined in the flow chart. The citation list is available upon request. |
| √ | Method of addressing articles published in languages other than English | Limits were placed to retrieve only English-language and human studies. |
| √ | Method of handling abstracts and unpublished studies | We had contacted a few authors for unpublished studies via e-mail but received no reply. |
| √ | Description of any contact with authors | We contacted authors who presented the outcome data in the form of figures instead of numerical values. We also requested for raw data for pretest-posttest correlation calculation. |
| **Reporting of methods should include** | | |
| √ | Description of relevance or appropriateness of studies assembled for assessing the hypothesis to be tested | Detailed inclusion and exclusion criteria were described in the methods section. |
| √ | Rationale for the selection and coding of data | A standardized form was used to record the data on the authors of each study, the year of publication, the country of origin, the sample size, age, sex, size of incision, influential factors, and outcome measures including the baseline and postoperative parameters. |
| √ | Assessment of confounding | Publication bias was assessed with Begg’s test (rank correlation method) and Egger’s test (linear regression method).  Conducted meta-regression to initially assess confounders.  Conducted subgroup analysis to further assess confounders.  Sensitivity analyses were also conducted by excluding one study at a time to evaluate the reliability of each study. |
| √ | Assessment of study quality, including blinding of quality assessors; stratification or regression on possible predictors of study results | Sensitivity analyses by several quality indicators such as timing of diabetes assessment relative to tuberculosis, method of diabetes and tuberculosis diagnosis, control selection, adjustment factors, potential duplicate data, use of convenience samples. |
| √ | Assessment of heterogeneity | The heterogeneity of the studies included was evaluated with the χ^2^ test and by examining the I^2^ value. |
| √ | Description of statistical methods in sufficient detail to be replicated | Description of methods of meta-analyses, sensitivity analyses, meta-regression and assessment of publication bias are detailed in the methods. |
| √ | Provision of appropriate tables and graphics | We included 1 flow chart, 4 forest plots, 3 supplementary tables illustrating details of each study included, and 1 supplementary table showing the result of meta-reguression. |
| **Reporting of results should include** | | |
| √ | Graph summarizing individual study estimates and overall estimate | Figure 2-5. |
| √ | Table giving descriptive information for each study included | Supplementary table 1-3 |
| √ | Results of sensitivity testing | In each part of result respectively. |
| √ | Indication of statistical uncertainty of findings | 95% confidence intervals were presented with all summary estimates, I^2^ values and results of sensitivity analyses |
| **Reporting of discussion should include** | | |
| √ | Quantitative assessment of bias | Publication bias was assessed with Begg’s test (rank correlation method) and Egger’s test (linear regression method).  Meta-regression, subgroup analysis and sensitivity analyses were used to indicate heterogeneity due to most common biases in observational studies. |
| √ | Justification for exclusion | We excluded studies that enrolled patients with systemic or ocular disease that may confound with dry eye evaluation. We also excluded studies that used topical or oral treatments that my influence tear stability / secretion. Low-quality studies that with conspicuous in the article or a lack of demographic information were also excluded. |
| √ | Assessment of quality of included studies | We discussed the results of the subgroup analysis, and potential reasons for the observed heterogeneity.  In the part of study limitations, sources of bias were further analyzed and evaluation quality was quantified. |
| **Reporting of conclusions should include** | | |
| √ | Consideration of alternative explanations for observed results | We discussed that multiple confounders could exist in the combination of outcomes. And study limitations also provide insights into possible factors that may interfere with dry eye outcomes. |
| √ | Generalization of the conclusions | According to our meta-analysis, cataract surgery does not induce or exacerbate DED in the general population. However, cataract patients with pre-existing MGD are more likely to suffer irritation symptoms, disrupted tear film stability, and a damaged corneal surface. Non-DM cataract patients are more susceptible to corneal nerve transection caused by incisions and display reduced reflex tearing after surgery compared with patients with DM. |
| √ | Guidelines for future research | We recommend clinical prediction of postoperative DED status based on preoperative DED evaluation of cataract patients. |
| √ | Disclosure of funding source | Funding source was stated in the acknowledgement. |
