# Supplementary figures and images for: Dry Eye and Phacoemulsification Cataract Surgery: A Systematic Review and Meta-Analysis
Source: Front Med (Lausanne). 2021 Jul 8;8:649030. doi: 10.3389/fmed.2021.649030 (PMC8295542; doi:10.3389/fmed.2021.649030)

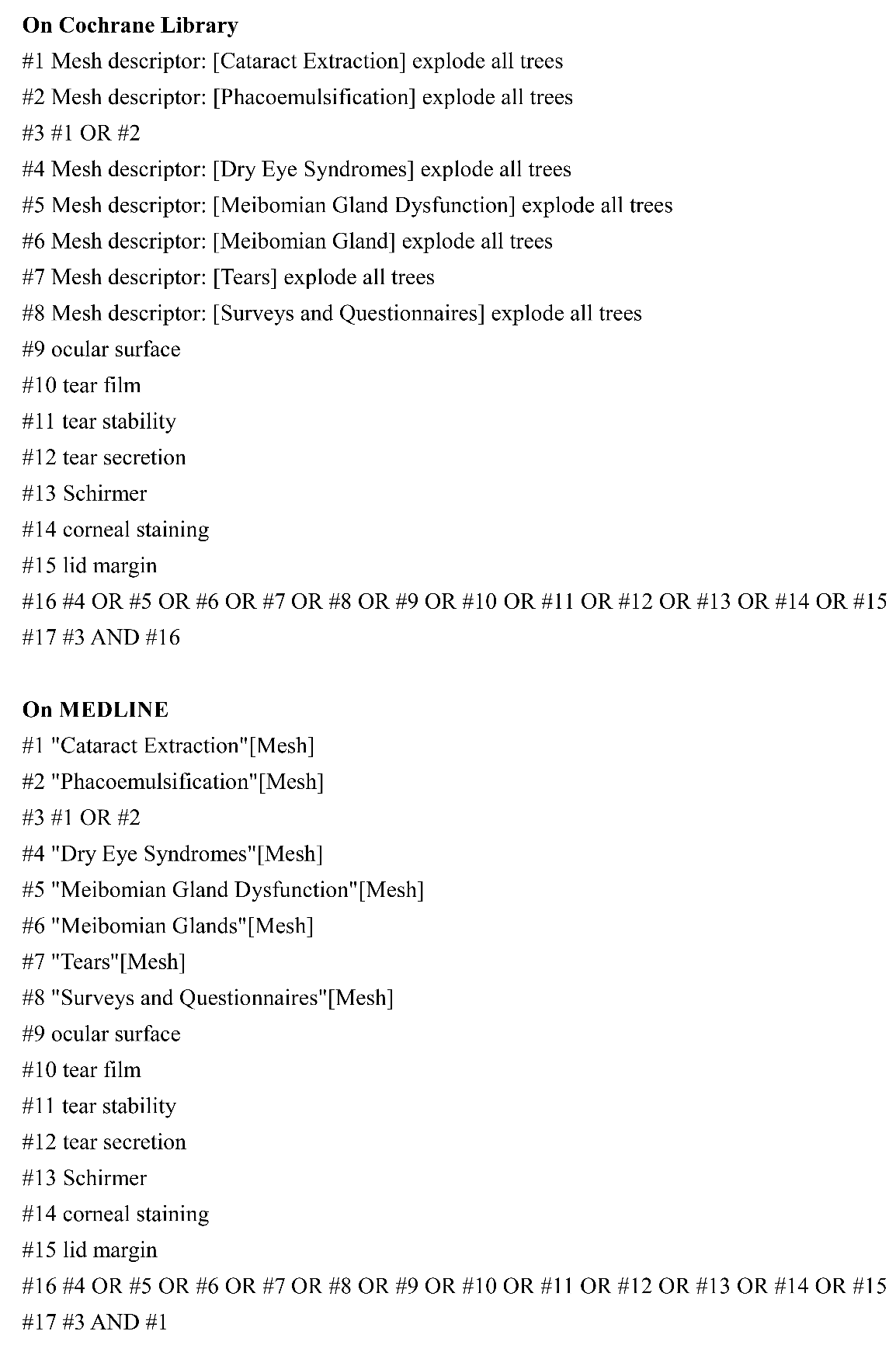

Supplement: Supplementary Figure 1 — Search strategies. [file Image_1.TIF]

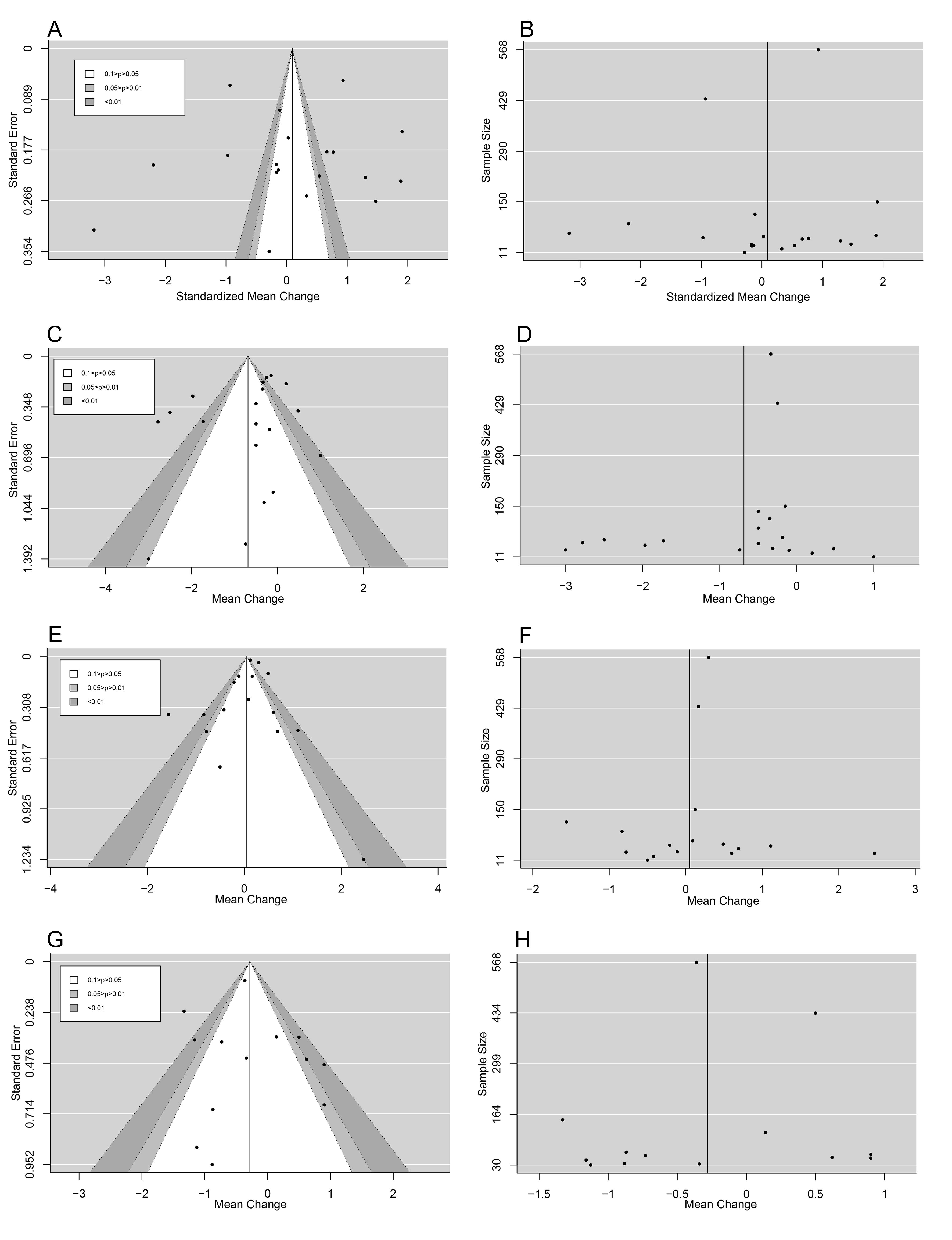

Supplement: Supplementary Figure 2 — Funnel plots for (A) dry eye questionnaires against standard error (SE); (B) dry eye questionnaires against sample size; (C) tear break-up time against SE; (D) tear break-up time against sample size; (E) corneal fluorescein staining score against SE (F) corneal fluorescein staining score against sample size; (G) Schirmer I test against SE; (H) Schirmer I test against sample size. [file Image_2.TIF]
