# Supplementary material for: Dry Eye and Phacoemulsification Cataract Surgery: A Systematic Review and Meta-Analysis
Source: Front Med (Lausanne). 2021 Jul 8;8:649030. doi: 10.3389/fmed.2021.649030 (PMC8295542; doi:10.3389/fmed.2021.649030)
Supplement: Supplementary Table 1 — Sensitivity analyses on dry eye questionnaires by excluding one study at a time. [file Table_3.DOCX]

Supplementary table 1. Sensitivity analyses on dry eye questionnaires by excluding one study at a time

| Exclusion | Sample size  (excluded/included) | Pooled effect size (95% CI)  (within subgroup) | I^2^ | Impact (*P*-value)  (within subgroup) ^a^ | Impact (*P*-value)  (between subgroups) ^a^ |
| --- | --- | --- | --- | --- | --- |
| Kyung Eun Han | 58 / 1857 | 1.0168 (0.5006, 1.5329) | 70.0367 | Remained (0.0001) | Remained (0.0049) |
| Ji Won Jung | 50 / 1865 | 1.5883 (1.0139, 2.1626) | 69.5964 | Remained (<0.0001) | Remained (<0.0010) |
| Jin Sun Kim | 43 / 1872 | 1.3175 (0.2260, 2.4089) | 93.0348 | Remained (0.018) | Remained (0.0230) |
| Donghong Jiang | 568 / 1347 | -0.2071 (-0.7616, 0.3474) | 97.7142 | Remained (0.4641) | Remained (0.0010) |
| Dewang Shao | 150 / 1765 | -0.2718 (-0.8092, 0.2656) | 98.2274 | Remained (0.3216) | Remained (0.0004) |
| Taehoon Oh | 48 / 1867 | -0.1880 (-0.7672, 0.3912) | 98.5178 | Remained (0.5246) | Remained (0.0012) |
| Yuli Park | 34 / 1881 | -0.2384 (-0.8048, 0.3281) | 98.4912 | Remained (0.4095) | Remained (0.0006) |
| MA Sanchez | 21 / 1894 | -0.1644 (-0.7392, 0.4104) | 98.5329 | Remained (0.5751) | Remained (0.0016) |
| Maierhaba Yusufu | 30 / 1885 | -0.1789 (-0.7551, 0.3974) | 98.5277 | Remained (0.5429) | Remained (0.0014) |
| Kensaku Miyake | 433 / 1482 | -0.0776 (-0.6187, 0.4636) | 97.721 | Remained (0.7787) | Remained (0.0028) |
| Maria Garcia Zamora | 55 / 1860 | -0.1454 (-0.7323, 0.4416) | 98.534 | Remained (0.6274) | Remained (0.0019) |
| Young Joon Choi | 116 / 1799 | -0.1363 (-0.7423, 0.4697) | 98.5283 | Remained (0.6593) | Remained (0.0020) |
| Hoseok Moon | 29 / 1886 | -0.1322 (-0.7099, 0.4455) | 98.5321 | Remained (0.6538) | Remained (0.0020) |
| Yinhui Yu | 64 / 1851 | 0.0599 (-0.4800, 0.5997) | 98.3488 | Remained (0.828) | Remained (0.0028) |
| A. El Ameen | 30 / 1885 | -0.1342 (-0.7123, 0.4440) | 98.5325 | Remained (0.6492) | Remained (0.0020) |
| Ana Gonzalez-Mesa | 52 / 1863 | -0.0777 (-0.6491, 0.4937) | 98.4815 | Remained (0.7899) | Remained (0.0028) |
| Do Yeh Yoon | 11 / 1904 | -0.1244 (-0.6953, 0.4464) | 98.5325 | Remained (0.6693) | Remained (0.0021) |
| Hun Lee | 33 / 1882 | -0.1318 (-0.7108, 0.4471) | 98.5317 | Remained (0.6554) | Remained (0.0020) |
| Ke Yao | 90 / 1825 | 0.0051 (-0.5342, 0.5445) | 98.3037 | Remained (0.9851) | Remained (0.0032) |

^a^ “Remained” stands for “remained statistically significant” or “remained not statistically significant”; “Changed” indicates that there was a shift from being statistically significant to being not statistically significantly or vice versa.

Supplementary table 2. Sensitivity analyses on tear break-up time by excluding one study at a time

| Exclusion | Sample size  (excluded/included) | Pooled effect size (95% CI)  (within subgroup) | I^2^ | Impact (*P*-value)  (within subgroup) ^a^ | Impact (*P*-value)  (between subgroups) ^a^ |
| --- | --- | --- | --- | --- | --- |
| Ji Won Jung | 50 / 1949 | -2.1479 (-2.5857, -1.7101) | 20.3525 | Remained (<0.0001) | Remained (<0.0001) |
| Jin Sun Kim | 43 / 1956 | -2.6185 (-3.1924, -2.0446) | 0 | Remained (<0.0001) | Remained (<0.0001) |
| Kyung Eun Han | 58 / 1941 | -2.1891 (-2.6479, -1.7303) | 57.6718 | Remained (<0.0001) | Remained (<0.0001) |
| A. El Ameen | 30 / 1969 | -0.2323 (-0.4479, -0.0167) | 45.1538 | Remained (0.0347) | Remained (<0.0001) |
| Dewang Shao | 150 / 1849 | -0.281 (-0.5532, -0.0087) | 52.1582 | Remained (0.0431) | Remained (<0.0001) |
| Do Yeh Yoon | 11 / 1988 | -0.2811 (-0.5034, -0.0589) | 46.8939 | Remained (0.0132) | Remained (<0.0001) |
| Donghong Jiang | 568 / 1431 | -0.2498 (-0.5095, 0.0100) | 51.6740 | Changed (0.0595) | Remained (<0.0001) |
| Hoseok Moon | 29 / 1970 | -0.2588 (-0.4942, -0.0235) | 52.6012 | Remained (0.0311) | Remained (<0.0001) |
| Hun Lee | 33 / 1966 | -0.2981 (-0.5249, -0.0712) | 46.2235 | Remained (0.0100) | Remained (<0.0001) |
| Ke Yao | 90 / 1909 | -0.2442 (-0.4834, -0.0051) | 51.9822 | Remained (0.0453) | Remained (<0.0001) |
| Kensaku Miyake | 433 / 1566 | -0.2652 (-0.5350, 0.0046) | 52.4795 | Changed (0.0540) | Remained (<0.0001) |
| MA Sanchez | 21 / 1978 | -0.3127 (-0.5444, -0.0810) | 41.9598 | Remained (0.0082) | Remained (<0.0001) |
| Maierhaba Yusufu | 30 / 1969 | -0.2522 (-0.4858, -0.0185) | 52.3522 | Remained (0.0344) | Remained (<0.0001) |
| Maria Garcia Zamora | 55 / 1944 | -0.1764 (-0.3484, -0.0043) | 21.4082 | Remained (0.0445) | Remained (<0.0001) |
| Rita Mencucci | 136 / 1863 | -0.2367 (-0.4794, 0.0060) | 51.2691 | Changed (0.0560) | Remained (<0.0001) |
| Taehoon Oh | 48 / 1951 | -0.2485 (-0.4856, -0.0115) | 52.2581 | Remained (0.0399) | Remained (<0.0001) |
| Yinhui Yu | 64 / 1935 | -0.2602 (-0.5002, -0.0202) | 52.6189 | Remained (0.0336) | Remained (<0.0001) |
| Young Joon Choi | 116 / 1883 | -0.248 (-0.5013, 0.0052) | 51.9467 | Changed (0.0549) | Remained (<0.0001) |
| Yuli Park | 34 / 1965 | -0.2557 (-0.4908, -0.0206) | 52.6080 | Remained (0.0330) | Remained (<0.0001) |

^a^ “Remained” stands for “remained statistically significant” or “remained not statistically significant”; “Changed” indicates that there was a shift from being statistically significant to being not statistically significantly or vice versa.

Supplementary table 3. Sensitivity analyses on corneal fluorescein staining by excluding one study at a time

| Exclusion | Sample size  (excluded/included) | Pooled effect size (95% CI)  (within subgroup) | I^2^ | Impact (*P*-value)  (within subgroup) ^a^ | Impact (*P*-value)  (between subgroups) ^a^ |
| --- | --- | --- | --- | --- | --- |
| Ji Won Jung | 50 / 1730 | 0.69 (-0.2031, 1.5831) | NA | Changed (0.1300) | Changed (0.2343) |
| Jin Sun Kim | 43 / 1737 | 1.11 (0.2298, 1.9902) | NA | Remained (0.0135) | Changed (0.0652) |
| Maria Garcia Zamora | 55 / 1725 | -0.0521 (-0.2173, 0.1131) | 83.7364 | Remained (0.5367) | Remained (0.0033) |
| Ana Gonzalez-Mesa | 52 / 1728 | 0.0356 (-0.1287, 0.1999) | 84.7513 | Remained (0.6710) | Remained (0.0068) |
| Dewang Shao | 150 / 1630 | -0.0783 (-0.3192, 0.1626) | 84.2831 | Remained (0.5239) | Remained (0.0052) |
| Yuli Park | 34 / 1746 | 0.0252 (-0.1426, 0.1930) | 84.8267 | Remained (0.7683) | Remained (0.0064) |
| MA Sanchez | 21 / 1759 | 0.0311 (-0.129, 0.1913) | 85.2200 | Remained (0.7034) | Remained (0.0072) |
| Yinhui Yu | 64 / 1716 | 0.003 (-0.1612, 0.1672) | 85.7726 | Remained (0.9716) | Remained (0.0054) |
| Donghong Jiang | 568 / 1212 | -0.0828 (-0.2978, 0.1323) | 81.4120 | Remained (0.4507) | Remained (0.0042) |
| A. El Ameen | 30 / 1750 | -0.0152 (-0.1763, 0.1459) | 85.4951 | Remained (0.8531) | Remained (0.0036) |
| Do Yeh Yoon | 11 / 1769 | 0.0166 (-0.1425, 0.1756) | 85.6201 | Remained (0.8384) | Remained (0.0064) |
| Kensaku Miyake | 433 / 1347 | -0.0158 (-0.1878, 0.1563) | 85.7848 | Remained (0.8576) | Remained (0.005) |
| Maierhaba Yusufu | 30 / 1750 | 0.0024 (-0.1539, 0.1587) | 85.1717 | Remained (0.9764) | Remained (0.0039) |
| Ke Yao | 90 / 1690 | 0.0491 (-0.1066, 0.2049) | 84.3114 | Remained (0.5363) | Remained (0.0083) |
| Hun Lee | 33 / 1747 | 0.0333 (-0.1243, 0.1909) | 85.0317 | Remained (0.6784) | Remained (0.0076) |
| Young Joon Choi | 116 / 1664 | 0.0895 (-0.0518, 0.2308) | 80.1959 | Remained (0.2143) | Remained (0.0108) |

^a^ “Remained” stands for “remained statistically significant” or “remained not statistically significant”; “Changed” indicates that there was a shift from being statistically significant to being not statistically significantly or vice versa.

NA = not applicable.

Supplementary table 4. Sensitivity analyses on Schirmer Ⅰ test by excluding one study at a time

| Exclusion | Sample size  (excluded/included) | Pooled effect size (95% CI)  (within subgroup) | I^2^ | Impact (*P*-value)  (within subgroup) ^a^ | Impact (*P*-value)  (between subgroups) ^a^ |
| --- | --- | --- | --- | --- | --- |
| Donghong Jiang | 568 / 1114 | -1.1267 (-2.8346, 0.5813) | NA | Changed (0.196) | vs subgroup2: Changed (0.8950) |
|  |  |  |  |  | vs subgroup3: Changed (0.1587) |
| Maierhaba Yusufu | 30 / 1652 | -0.3611 (-0.5351, -0.1871) | NA | Remained (<0.0001) | vs subgroup2: Remained (0.0047) |
|  |  |  |  |  | vs subgroup3: Changed (0.1181) |
| Dewang Shao | 150 / 1532 | -1.0892 (-1.7148, -0.4635) | 0 | Remained (0.0006) | vs subgroup1: Remained (0.0297) |
|  |  |  |  |  | vs subgroup3: Remained (0.0013) |
| Jin Sun Kim | 43 / 1639 | -1.2774 (-1.7055, -0.8493) | 0 | Remained (<0.0001) | vs subgroup1: Remained (0.0001) |
|  |  |  |  |  | vs subgroup3: Remained (<0.0001) |
| Yinhui Yu | 64 / 1618 | -1.2815 (-1.6660, -0.8969) | 0 | Remained (<0.0001) | vs subgroup1: Remained (<0.0001) |
|  |  |  |  |  | vs subgroup3: Remained (<0.0001) |
| Hun Lee | 33 / 1649 | 0.2198 (-0.1094, 0.5491) | 50.5953 | Remained (0.1907) | vs subgroup1: Remained (0.0207) |
|  |  |  |  |  | vs subgroup2: Remained (<0.0001) |
| Ji Won Jung | 50 / 1632 | 0.0892 (-0.2395, 0.4179) | 51.2440 | Remained (0.5947) | vs subgroup1: Changed (0.0793) |
|  |  |  |  |  | vs subgroup2: Remained (<0.0001) |
| Kensaku Miyake | 433 / 1249 | 0.0661 (-0.2785, 0.4108) | 51.1626 | Remained (0.7068) | vs subgroup1: Changed (0.0880) |
|  |  |  |  |  | vs subgroup2: Remained (<0.0001) |
| Kyung Eun Han | 58 / 1624 | 0.1085 (-0.2090, 0.4260) | 50.7380 | Remained (0.5028) | vs subgroup1: Changed (0.0643) |
|  |  |  |  |  | vs subgroup2: Remained (<0.0001) |
| Maria Garcia Zamora | 55 / 1627 | 0.3389 (-0.0009, 0.6787) | 12.3128 | Remained (0.0506) | vs subgroup1: Changed (0.0003) |
|  |  |  |  |  | vs subgroup2: Remained (<0.0001) |
| Taehoon Oh | 48 / 1634 | 0.0633 (-0.2631, 0.3897) | 44.5228 | Remained (0.7039) | vs subgroup1: Changed (0.0817) |
|  |  |  |  |  | vs subgroup2: Remained (<0.0001) |
| Young Joon Choi | 116 / 1566 | 0.1550 (-0.1900, 0.5000) | 55.5191 | Remained (0.3786) | vs subgroup1: Changed (0.0592) |
|  |  |  |  |  | vs subgroup2: Remained (<0.0001) |
| Yuli Park | 34 / 1648 | 0.1811 (-0.1319, 0.4940) | 51.1271 | Remained (0.2568) | vs subgroup1: Changed (0.0187) |
|  |  |  |  |  | vs subgroup2: Remained (<0.0001) |

^a^ “Remained” stands for “remained statistically significant” or “remained not statistically significant”; “Changed” indicates that there was a shift from being statistically significant to being not statistically significantly or vice versa.

NA = not applicable.
